# Beyond the Dispensary: Pharmacists’ Perspectives on the Responsible Implementation of Emerging Clinical, Digital, and Ethically Sensitive Roles

**DOI:** 10.3390/pharmacy14060140

**Published:** 2026-09-21

**Authors:** Christopher Mosch, Clara Simon, Enes Dusinovic, Stephanie Clemens, Johanna Pachmayr, Olaf Rose

**Affiliations:** 1 Institute of Pharmacy, Pharmaceutical Biology and Clinical Pharmacy, Paracelsus Medical Private University, Strubergasse 21, 5020 Salzburg, Austria; christopher.mosch@stud.pmu.ac.at (C.M.); enes.dusinovic@teach.pmu.ac.at (E.D.);; 2 Center of Public Health and Health Services Research, Paracelsus Medical Private University, Strubergasse 15, 5020 Salzburg, Austria

**Keywords:** pharmacy practice, implementation, assisted suicide, point-of-care testing, artificial intelligence, mixed methods, pharmaceutical care

## Abstract

Pharmacy practice is expanding into clinical, digital, and ethically sensitive areas, yet professional support may not translate into implementation. Building on previous studies in point-of-care testing (POCT), artificial intelligence (AI), and assisted suicide, this exploratory convergent mixed-methods study used a purpose-designed symposium workshop focused on responsible implementation. Participants completed an anonymous 15-item questionnaire and submitted anonymous online barrier entries during the workshop. Of 45 returned questionnaires, 42 met the predefined completeness criterion for inferential analyses; 186 barrier entries were analyzed using structured qualitative content analysis. POCT showed high perceived capability, relevance, support, and willingness, while barriers centered on resources and economic viability. AI was considered highly relevant, but perceived capability was lower and concerns focused on reliability, regulation, accountability, and uncritical use. Pharmacist involvement in assisted suicide was broadly supported, while perceived capability was lower and barriers spanned ethical, organizational, legal, communicative, and emotional domains. These field-specific patterns were heuristically summarized as endorsement-capacity, capability-governance, and support-preparedness gaps. The labels are descriptive, non-validated summaries rather than participant-level mismatches. The findings suggest that professional support alone is an insufficient indicator of implementation readiness, as each activity was characterized by a distinct combination of capability, organizational capacity, and governance or support needs.

## 1. Introduction

Pharmacy practice is expanding beyond established medicines-focused responsibilities into broader roles in prevention, clinical assessment, medication management, digital decision support, and ethically complex care. This development is reflected in new services, changing legal responsibilities, and growing expectations that pharmacists contribute directly to patient pathways and population health [1,2]. However, the scope and implementation of community pharmacy services vary substantially across jurisdictions: medication review, pharmacist prescribing, vaccination, screening, referral, and other patient-centered clinical pharmacy services may be established, emerging, or restricted [3].

An expanded scope of practice is not implemented simply because a service is legally permitted, technically possible, or professionally desirable. Responsible implementation requires several conditions to align: the role must be regarded as legitimate and relevant; professionals must feel sufficiently prepared; organizations must have time, staff, infrastructure, and financing; and the service must be supported by quality assurance, accountability, referral pathways, and appropriate ethical safeguards [4,5].

Implementation also depends on sufficient public acceptability. Surveys that assess only pharmacists’ general acceptance of new services may therefore overestimate practical readiness. Established implementation science distinguishes multilevel determinants from implementation outcomes such as acceptability, appropriateness, feasibility, adoption, fidelity, cost, and sustainability, as well as from organizational readiness [6,7,8]. These concepts informed the interpretation of the present study but were not operationalized as validated scales or as a formal readiness framework.

Point-of-care testing (POCT), artificial intelligence (AI), and assisted suicide illustrate three different boundaries of contemporary pharmacy practice. POCT, here referring to standardized rapid testing performed in community pharmacies, extends pharmacists’ clinical and preventive function through testing, interpretation, counselling, and referral without implying medical diagnosis [5,6,7,8,9]. AI-powered clinical decision-support tools introduce digital support into medication review and pharmacotherapy while raising questions about validation, critical appraisal, human oversight, and professional responsibility [9,10]. Assisted suicide places pharmacists within a legally regulated but morally, ethically and emotionally demanding process in which professional duties, voluntary participation, communication, and support structures require particular attention [11,12,13].

In Austria, following medical counselling and eligibility assessment and establishment of a legally valid declaration of intent for assisted suicide (Sterbeverfügung) before a notary or another authorized legal professional, willing community pharmacies may dispense the specified lethal preparation together with the required adjunct medication [13]. At dispensing, pharmacists verify the identity of the collecting person and the applicable register status, document dispensing, and provide practical medication-related counselling on handling, administration, expected effects, and potential complications. The lethal preparation is self-administered by the patient.

The three fields differ markedly in technical maturity, ethical sensitivity, and organizational requirements, making them useful contrasting cases for examining the responsible expansion of pharmacy practice. They also involve different forms of professional responsibility: appropriate interpretation and referral in POCT, critical appraisal and human oversight in AI, and lawful, voluntary, and ethically supported participation in assisted suicide.

The regulatory context also varies internationally. In Austria, POCT and medication review lack routine reimbursement, routine independent pharmacist prescribing is not authorized, and vaccination remains restricted to physicians [14]. In Germany, pharmacists are reimbursed for medication review and are remunerated for vaccinations [15].

Under the “pharmacy first program”, British pharmacists serve as accessible healthcare navigators, guiding patients to appropriate services and levels of care [16]. In parts of Canada, pharmacists can manage chronic diseases and initiate or order laboratory tests within their authorized scope of practice [17]. Assisted-dying supply arrangements also differ: Austria permits dispensing by willing community pharmacies, whereas Western Australia uses a designated statewide pharmacy service [18,19]. These differences in legal scope, service maturity, remuneration, and professional roles limit the direct transferability of findings between jurisdictions [13].

Existing research has largely examined these activities within separate topic-specific studies and regulatory contexts. One such topic-specific investigation was the previously published PAS study, which used national online surveys and semi-structured interviews to examine Austrian community pharmacists’ perspectives on assisted suicide, including attitudes, experience, perceived mental capability, barriers, and support strategies [13]. Consequently, it remains unclear to what extent barriers identified across different areas of pharmacy practice reflect common challenges of professional expansion or requirements specific to the clinical, digital, or ethical nature of a particular role. Examining deliberately contrasting activities within a single professional event sample and a common deliberative setting allows these patterns to be compared under a shared analytic perspective while retaining the topic-specific meaning of the individual measures.

The symposium workshop was conceived as a deliberate next step beyond these topic-specific investigations, moving from the description of attitudes and barriers toward discussion of the conditions, priorities, and safeguards required for responsible implementation. The present manuscript analyzes an entirely separate dataset generated during this symposium workshop; no previously collected POCT, AI, assisted-suicide survey, interview, or other participant-level data were reused. As no cross-study identifiers were available, possible overlap of individual participants with previous studies cannot be determined. The specific novel contribution of the present study therefore lies not in providing another population estimate for any single field, but in the side-by-side comparison of POCT, AI, and assisted-suicide-related practice and the field-level integration of structured ratings with submitted implementation barriers to identify priorities for the continued development of these roles. The approach is exploratory and hypothesis-generating and does not seek to validate a general implementation or readiness framework.

This study therefore examined pharmacists’ perspectives on POCT, AI, and assisted suicide within an interactive professional symposium. It aimed to: (1) describe perceived capability, relevance, support, field-specific risk, and implementation-related willingness for each field; (2) identify the implementation barriers participants associated with each field; and (3) integrate these findings to characterize field-specific implementation patterns and generate hypotheses for priorities in the continued development of pharmacy practice. A prespecified quantitative hypothesis was that both POCT and AI would be rated as more relevant to pharmacists than assisted suicide.

## 2. Materials and Methods

### 2.1. Study Design and Setting

A single-event, non-interventional, cross-sectional convergent mixed-methods study was conducted during a purpose-designed interactive symposium workshop devoted to the responsible implementation of emerging pharmacy roles in Salzburg, Austria, on 5 June 2026. The workshop was deliberately conceived as a next-step format building on previous studies conducted by our research group in POCT, AI-supported pharmacotherapy, and assisted suicide [10,13,20], and shifted the focus from field-specific findings toward considering how these emerging roles could be developed and implemented responsibly. The workshop combined introductory presentations and moderated discussions led by researchers from pharmacy, medicine, and nursing; a quantitative strand comprising an anonymous paper questionnaire; and a qualitative strand comprising anonymous online free-text prompts. The educational and deliberative setting was an intentional component of the design; the study sought informed judgments following topic-specific scientific input and professional discussion rather than unconditioned baseline attitudes. It was not designed as a formal consensus or Delphi process.

The questionnaire and online entries constituted the two formal data strands; presentations, live visualization, and moderated discussions provided the deliberative context but were not analyzed as an independent qualitative dataset. Questionnaire responses and free-text entries were analyzed separately and subsequently integrated at the topic level. The quantitative strand enabled item-level descriptions and comparisons, whereas the qualitative strand was used to identify and classify perceived implementation barriers. The prespecified analyses described the five questionnaire items by field, tested whether POCT and AI were rated as more relevant than assisted suicide, explored associations of prior experience with perceived capability or willingness, and classified the anonymous barrier entries. Quantitative and qualitative responses were anonymous and could not be linked at participant level.

### 2.2. Participants and Recruitment

Participants were recruited through a national professional announcement circulated by the Austrian Chamber of Pharmacists. The event was openly advertised as an interactive symposium workshop combining scientific presentations, moderated discussion, and study participation as integral components of the same format. Registration was available through the event website. All pharmacists attending the event were eligible to participate in the study, and all took part in the combined symposium and workshop activities. Country of employment was not an exclusion criterion; pharmacists working outside Austria could therefore participate if they attended the Austrian-hosted event. Attendance at the event was self-selected, and the resulting sample represented a professionally engaged, predominantly Austrian group rather than a representative sample of Austrian pharmacists. Study information was provided on site, and consent was indicated by voluntary completion and return of the anonymous questionnaire or submission of an online entry.

### 2.3. Workshop Sequence and Data Collection

The three fields were addressed in separate thematic blocks during the workshop. Each block comprised a brief topic-specific scientific presentation followed by moderated discussion with the participating pharmacists. Anonymous online barrier entries were subsequently elicited via Mentimeter (Mentimeter AB, Stockholm, Sweden; accessed on 5 June 2026) and visualized live; the displayed responses could then be taken up in a concluding moderated discussion. The paper questionnaire had been distributed at the beginning of the workshop and was collected at the end; individual item-completion times were not recorded. The questionnaire captured individual ratings of perceived capability, relevance, support, field-specific risk, and implementation-related willingness. The online prompts elicited brief, spontaneous statements about perceived barriers.

### 2.4. Questionnaire

The study-specific questionnaire comprised 15 closed items, with five items addressing each of the three fields POCT, AI, and assisted suicide, supplemented by questions on demographic and professional characteristics and prior field-specific experience (Table S1).

Five broad content areas were addressed within each field: perceived capability, professional relevance, support for pharmacist involvement, field-specific risk, and implementation-related willingness. The items were developed from content areas prespecified in the dated study protocol and underwent internal face and content review and pretesting before data collection [21,22].

Three authors (C.M., E.D., and C.S.) reviewed the items within the PMU Pharmacotherapy Research Group. They had pharmacy and pharmacotherapy expertise and familiarity with the three workshop topics and the intended professional audience. The review considered apparent relevance and coverage of the prespecified content areas, appropriateness for the intended audience, clarity, readability, comprehensibility, response-option fit, layout, and practical administration [22,23].

Feedback resulted in minor wording and layout refinements; the predefined content areas, four-point response scale, and scoring remained unchanged. This fit-for-purpose procedure provided an internal qualitative assessment of face and content validity but did not constitute a formal quantitative content-validity study or psychometric validation.

The perceived-capability items assessed whether respondents felt able to perform POCT, interpret the results, and counsel patients appropriately; use AI in medication reviews and appropriately interpret its outputs; or undertake assisted-suicide-related tasks professionally and in accordance with the applicable legal requirements. The term perceived capability is used throughout this manuscript rather than self-efficacy, because each field was assessed using a single study-specific item rather than a validated multi-item self-efficacy scale. The remaining items assessed professional relevance, support for pharmacist involvement, field-specific risk, and implementation-related willingness. Risk referred to false reassurance after POCT, uncritical adherence to AI recommendations, and emotional burden in assisted-suicide-related practice; willingness referred to provision of POCT, intention or prior use of AI in medication reviews, and willingness to undertake assisted-suicide-related tasks.

Responses were recorded on a four-point Likert-like scale ranging from 1 (strongly disagree) to 4 (strongly agree). The neutral midpoint was deliberately omitted to elicit a directional judgment within the deliberative workshop context rather than a neutral baseline position. Agreement was defined as response options 3 or 4; all ordinal analyses retained the original four categories. Because each content area was represented by a single item within each field, no internal-consistency coefficient was calculated. The items assessed individual perceptions and intentions and were not intended to estimate latent constructs, organizational readiness, feasibility, adoption, fidelity, sustainability, or patient outcomes.

### 2.5. Online Barrier Prompts

Three anonymous online prompts asked participants to identify perceived barriers to the implementation or practical application of POCT, AI in pharmacotherapy, and assisted-suicide-related services. The prompts were administered using Mentimeter Pro, a web-based audience-response platform. The prompts were open-ended, allowed multiple submissions, and captured immediate implementation concerns rather than interview-depth narratives. The unit of analysis was an individual entry; repeated submissions were retained, and frequencies therefore describe entries rather than participant-level prevalence. Because entries were anonymous, unlinked, and could not be probed, integration was limited to the aggregate field level and no thematic saturation was claimed.

### 2.6. Data Processing and Quality Assurance

Questionnaire responses were entered into Microsoft Excel (version 2607; Microsoft Corporation, Redmond, WA, USA). Ambiguous or uninterpretable markings were treated as item-level missing values. Descriptive analyses used all valid responses and item-specific denominators; questionnaires with at least 12 of 15 completed items were included in inferential analyses. Missing values were not imputed. Online entries were retained as separate records in accordance with the Mentimeter export. Repeated identical submissions were not collapsed. Spelling variants and semantically equivalent expressions were considered during qualitative coding but remained unchanged in the source data. The anonymized questionnaire dataset and coded online-entry dataset were checked for data consistency. Reported counts, denominators, percentages, test statistics, multiplicity adjustments, and effect sizes were independently checked by another author against the corresponding source data and analysis outputs.

### 2.7. Quantitative Analysis

Counts, percentages, medians, interquartile ranges, means, and standard deviations were calculated for each questionnaire item. Percentages were based on item-specific valid denominators. Risk items were analyzed in their original direction, such that higher scores indicated greater agreement with the stated risk. For visual presentation of favorable response profiles, disagreement with the negatively worded risk items was presented so that higher percentages consistently represented a more favorable profile.

Within-participant comparison of relevance ratings across the three fields was conducted using the Friedman test, with Kendall’s W reported as an effect-size measure. This was the sole prespecified principal cross-field inferential comparison. Perceived capability, support, field-specific risk, and implementation-related willingness were summarized descriptively across fields because the corresponding items were topic-specific, not fully parallel, and were not intended as measurement-equivalent indicators of common latent constructs.

The overall relevance comparison was followed by three Wilcoxon signed-rank contrasts: POCT versus AI, POCT versus assisted suicide, and AI versus assisted suicide. These three contrasts constituted one family and were adjusted using the Holm–Bonferroni method. Associations between prior field-specific experience and perceived capability or implementation-related willingness were examined using Mann–Whitney U tests, with Cliff’s delta reported as an effect-size measure. The six comparisons, comprising two outcomes for each of the three fields, constituted a separate exploratory family and were adjusted using the Holm-Bonferroni method. Complete paired observations were used for each repeated-measures analysis. All tests were two-sided, with α = 0.05. Analyses were implemented in Python 3.11. For reproducibility, a dependency-free script compatible with Python 3.11 or later and verified with Python 3.12.14 is provided in the repository.

### 2.8. Qualitative Content Analysis

The online entries were analyzed at the level of manifest content using structured qualitative content analysis with combined deductive-inductive category development [24]. Two authors (E.D. and C.S.) independently categorized all 186 entries in separate preliminary coding workbooks and refined initial categories against the material. E.D., C.S., and C.M. then compared and harmonized the preliminary category systems into a shared 11-category codebook. Each entry was assigned to one predominant category, while retaining its field context. Semantic variants were coded consistently, and repeated submissions remained separate contributions. The harmonized codebook and all final category assignments were manually reviewed by E.D., C.S., and C.M.; ambiguous or boundary cases were discussed until consensus was reached. No inter-rater reliability coefficient was calculated because the final classifications were consensus decisions and the two preliminary coders had not independently applied the same finalized codebook. Primary-category frequencies and percentages were summarized by field. Given the brevity and non-probed nature of the entries, interpretation was limited to manifest content and no thematic saturation was claimed.

### 2.9. Mixed-Methods Integration

The quantitative and qualitative strands were analyzed separately before integration. Integration was performed at the field level by placing the questionnaire findings and primary barrier-category profiles side by side for POCT, AI, and assisted suicide in a joint display [25]. The integrated interpretation considered convergence, complementarity, and apparent divergence between perceived capability, professional relevance, support, field-specific risk, implementation-related willingness, and the barriers expressed in the online entries. This approach was used to characterize the aggregate pattern within each field by considering professional endorsement alongside the conditions perceived as necessary for responsible implementation. Live visualization and discussion provided interpretive context but were not analyzed as a separate qualitative strand. Integration was limited to field level because the datasets were anonymous and unlinked; the field-specific gap labels were developed post hoc as nonvalidated interpretive summaries.

### 2.10. Ethics

The study was conducted in accordance with the Declaration of Helsinki and received approval from the Institutional Ethics Committee of Paracelsus Medical Private University (PMU-EK-2026-0030) [26]. Participation was voluntary. Consent was indicated by completion and return of the anonymous paper questionnaire or by voluntary submission of an online entry. No names, contact details, or other direct personal identifiers were collected. Questionnaire responses and online entries were not linked.

## 3. Results

### 3.1. Participant Characteristics and Data Completeness

All 45 participants returned questionnaires; 42 met the 80% threshold for inferential analyses, while three contributed valid item-level data only (denominators 42–45). Questionnaire return among participants was therefore complete. Most worked in community pharmacies (80.0%) and Austria (82.2%); 55.5% had more than 10 years of professional experience. Prior field experience was reported for POCT (62.2%), AI (15.6%), and assisted suicide (26.7%) (Table 1).

Item-level questionnaire results across the three fields are presented in Table 2.

**Figure 1:**
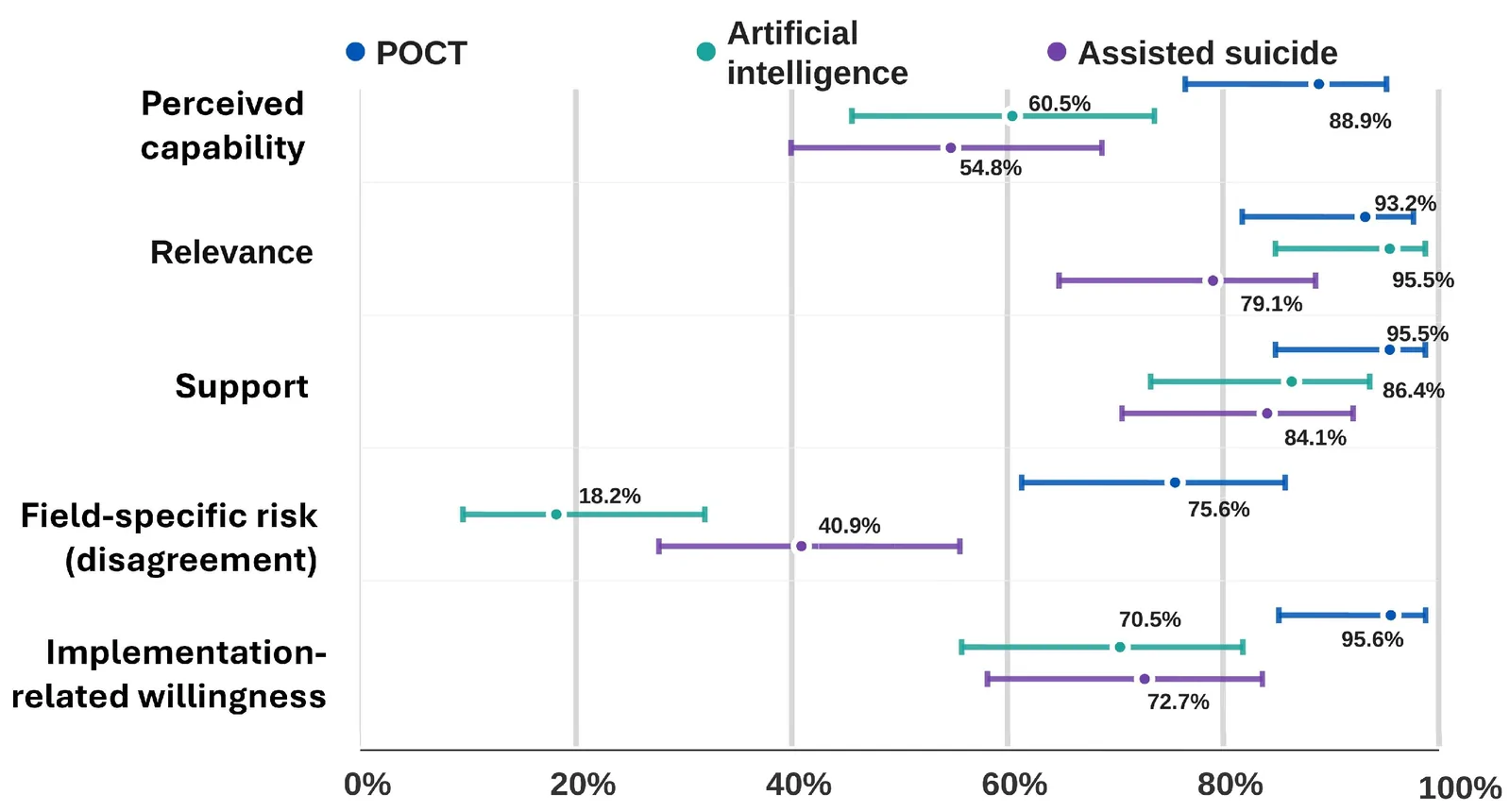
Favorable response profiles for point-of-care testing, artificial intelligence, and assisted suicide across perceived capability, relevance, support, field-specific risk, and implementation-related willingness. For the negatively worded field-specific risk items, disagreement is displayed so that higher values consistently indicate a more favorable profile. Error bars represent 95% Wilson confidence intervals for the displayed proportions.

The corresponding favorable response profiles with 95% Wilson confidence intervals are visualized in Figure 1. For the negatively worded risk items, disagreement is displayed so that higher values consistently indicate a more favorable profile.

**Figure 2:**
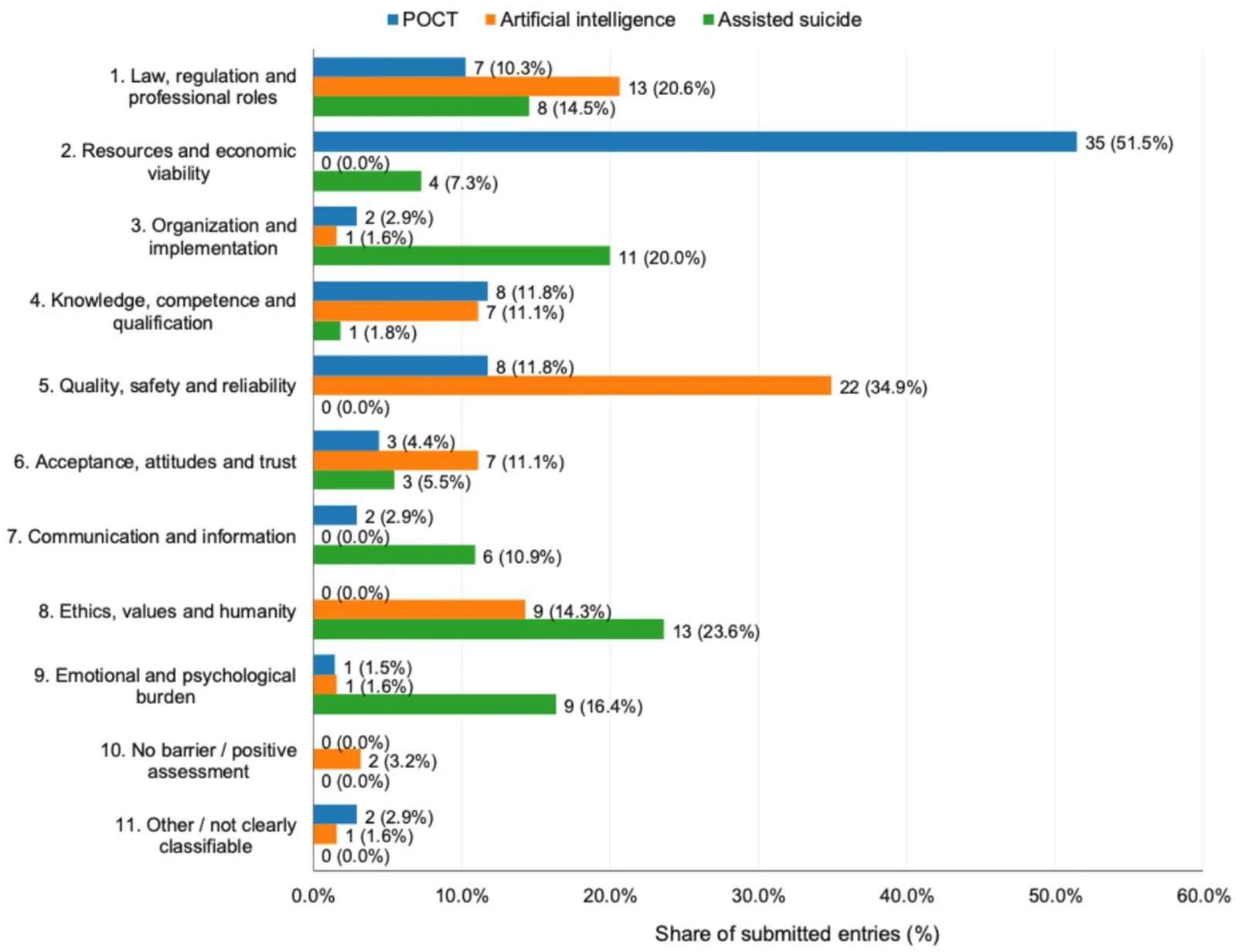
Primary barrier-category profiles for point-of-care testing, artificial intelligence, and assisted suicide. Percentages are based on topic-specific entry denominators and refer to anonymous entries rather than respondents.

Participants submitted 186 barrier entries (POCT, 68; AI, 63; assisted suicide, 55). Frequencies describe entries rather than respondents. The distribution of barrier categories differed markedly across the three fields. Resources and economic viability predominated for POCT, quality, safety, and reliability for AI, whereas the barriers associated with assisted suicide were distributed more broadly across ethical, organizational, emotional, legal, and communicative domains. These contrasting profiles are visualized in Figure 2.

### 3.2. Point-of-Care Testing: Strong Endorsement but Limited Delivery Capacity

POCT received consistently favorable ratings: capability 88.9%, relevance 93.2%, support 95.5%, and implementation-related willingness 95.6%; 24.4% agreed with the stated risk (Table 2). Resources and economic viability dominated the POCT entries (35/68; 51.5%), particularly time, staffing, remuneration, space, and equipment. Quality, safety, reliability and knowledge, competence, and qualification each accounted for 8/68 entries (11.8%); remaining categories were less frequent. Thus, strong endorsement contrasted primarily with concerns about sustainable integration into routine practice.

### 3.3. Artificial Intelligence: High Relevance with Capability and Governance Concerns

AI was considered relevant by 95.5% and supported by 86.4%, whereas perceived capability (60.5%) and implementation-related willingness or prior use (70.5%) were lower. Concern about uncritical adherence was reported by 81.8% (Table 2).

Quality, safety, and reliability dominated AI barriers (22/63; 34.9%), followed by law, regulation, and professional roles (13/63; 20.6%). Entries concerned inaccurate or outdated outputs, data security, standards, responsibility, liability, and human decision authority. Ethics, values, and humanity accounted for 9/63 entries (14.3%), while knowledge, competence, and qualification and acceptance, attitudes, and trust each accounted for 7/63 (11.1%). Remaining categories were infrequent. Overall, participants identified concerns regarding the conditions for valid, critical, and accountable use under meaningful human oversight.

### 3.4. Assisted Suicide: Professional Support Exceeded Perceived Preparedness

Assisted suicide was rated as relevant by 79.1%, supported by 84.1%, and associated with implementation-related willingness by 72.7%; perceived capability was 54.8%, and 59.1% agreed with the emotional-burden risk item (Table 2). Barriers were led by ethics, values, and humanity (13/55; 23.6%), organization and implementation (11/55; 20.0%), and emotional and psychological burden (9/55; 16.4%). Entries concerned moral conflict, team and management involvement, time and privacy, procedures, and emotional support. Law, regulation, and professional roles accounted for 8/55 entries (14.5%) and communication and information for 6/55 (10.9%); other categories were less frequent. Accordingly, the submitted barriers extended beyond medicine supply to legal, organizational, communicative, ethical, and emotional preparedness.

### 3.5. Cross-Field Comparisons and Prior Experience

The prespecified relevance comparison indicated a difference across the three fields (Friedman χ^2^ = 16.22, *p* < 0.001, Kendall’s W = 0.193; *n* = 42). Perceived capability, support, field-specific risk, and implementation-related willingness were retained as descriptive cross-field profiles only because the corresponding items were not fully parallel (Table 2; Figure 1).

The prespecified relevance hypothesis was partly supported. POCT was rated as more relevant for pharmacists than assisted suicide (Holm-adjusted *p* = 0.003) and more relevant than AI (Holm-adjusted *p* = 0.029). The difference between AI and assisted suicide did not meet the adjusted significance threshold (Holm-adjusted *p* = 0.083). These relative comparisons should be interpreted alongside the high absolute relevance ratings observed in all three fields.

Prior field-specific experience was not significantly associated with perceived capability or implementation-related willingness after adjustment for multiple testing. Before adjustment, perceived AI capability was higher among respondents with prior AI experience (*n* = 6; median 4.0) than among those without such experience (*n* = 36; median 3.0; unadjusted *p* = 0.012; Cliff’s delta = 0.63; Holm-adjusted *p* = 0.070). Perceived POCT capability was also higher among respondents with prior POCT experience (*n* = 28; median 4.0) than among those without such experience (*n* = 14; median 3.5; unadjusted *p* = 0.027; Cliff’s delta = 0.35; Holm-adjusted *p* = 0.134).

The cross-sectional design cannot determine whether experience increased perceived capability, whether participants with greater confidence were more likely to acquire experience, or whether both were related to other characteristics.

### 3.6. Mixed-Methods Integration: Three Field-Specific Implementation Patterns

Integration of the questionnaire and qualitative barrier data did not reveal a single general pattern across the three fields. Instead, each field showed a distinct aggregate combination of item responses and submitted implementation barriers. Because the quantitative and qualitative datasets were anonymous and unlinked, these patterns do not establish participant-level mismatches.

For POCT, high relevance, support, perceived capability, and implementation-related willingness coexisted with a qualitative profile dominated by staffing, time, financing, space, equipment, and workflow requirements. This aggregate pattern was heuristically summarized as an endorsement-capacity gap, with strong professional endorsement alongside resource and delivery-capacity concerns.

For AI, high relevance and support coexisted with less consistent perceived capability, substantial concern about uncritical use, and qualitative barriers involving reliability, validation, accountability, regulation, and human control. This aggregate pattern was heuristically summarized as a capability-governance gap, with submitted responses highlighting capability and governance arrangements as candidate implementation considerations.

For assisted suicide, support for pharmacist involvement and implementation-related willingness exceeded perceived capability, while ethical conflict, organizational requirements, legal uncertainty, communication, and emotional burden remained prominent. This aggregate pattern was heuristically summarized as a support-preparedness gap, with broad support alongside legal, organizational, communicative, ethical, and emotional preparedness concerns.

**Figure 3:**
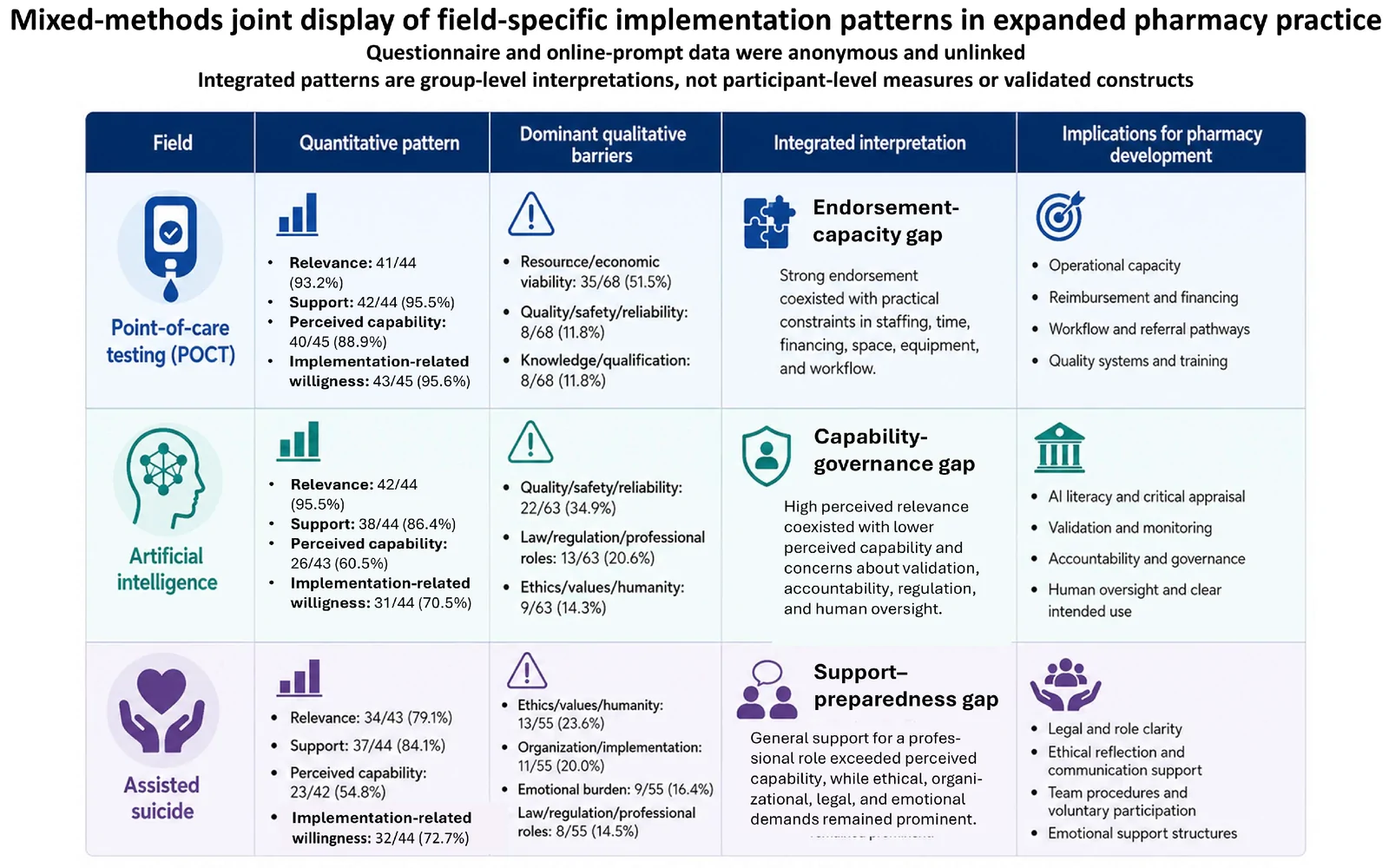
The joint display summarizes activity-specific patterns across professional legitimacy, capability, delivery capacity, and governance or supportive safeguards; the dominant pattern differed across the three fields.

These post hoc labels are heuristic, aggregate-level, non-validated interpretations; anonymous, unlinked datasets preclude attributing endorsement and barriers to the same individuals. They are descriptive shorthand for field-specific patterns, not measured constructs, participant-level mismatches, or a validated readiness framework (Table 3). Figure 3 provides a joint display integrating the quantitative response profiles, dominant qualitative barriers, and field-specific implementation considerations.

## 4. Discussion

Across all three fields, participants expressed generally favorable attitudes, although the perceived barriers and conditions for implementation differed substantially between POCT, AI, and assisted suicide. In this sense, the symposium workshop represented a progression from documenting field-specific attitudes and barriers toward considering what would be required to move these emerging roles into responsible practice. POCT was constrained primarily by delivery capacity, AI by capability and governance, and assisted suicide by professional, organizational, ethical, legal, communicative, and emotional preparedness. The study’s principal contribution is therefore comparative: support for an expanded role does not itself establish readiness, and the transition to practice differs by activity. These field-specific patterns can be summarized heuristically as endorsement-capacity, capability-governance, and support-preparedness gaps. The labels are descriptive shorthand rather than validated constructs and are intended to generate hypotheses for future implementation research. The patterns suggest that a uniform implementation strategy is unlikely to address the different concerns identified across the three activities. Training cannot compensate for an unfunded POCT service; access to AI cannot resolve validation and accountability; and legal authorization cannot alone prepare pharmacy professionals for assisted-suicide-related work. The patterns therefore suggest that future implementation research should examine field-specific combinations of professional legitimacy, individual capability, organizational delivery capacity, and governance or supportive safeguards. Live visualization and moderated discussion supported collective contextualization of the emerging findings. Because these discussions were not systematically documented or analyzed, they provide interpretive context rather than evidence of formal consensus.

### 4.1. Point-of-Care Testing: Converting Professional Endorsement into Service Capacity

POCT combined high support, perceived capability, and willingness with barriers dominated by resources and economic viability. The principal challenge therefore appears to be sustainable delivery within routine pharmacy operations rather than professional acceptance.

Responsible POCT requires an integrated service model encompassing staffing, protected time, space, consumables, maintenance, quality control, documentation, interpretation, data protection, communication, and referral pathways. Remuneration must cover professional time and quality assurance as well as the test itself, as highlighted in a recent scoping review of community-pharmacy POCT for antimicrobial stewardship [27]. Without these conditions, high willingness may coexist with low implementation capacity.

Future evaluations should therefore test complete POCT care pathways, including eligibility, downstream decisions, false results, referral, feasibility, cost-effectiveness, and patient outcomes [28]. Relevant implementation outcomes extend beyond attitudes to adoption, fidelity, reach, cost, sustainability, and subsequent care.

### 4.2. Artificial Intelligence: Moving from Perceived Relevance to Critically Governed Use

AI combined near-universal relevance with lower perceived capability and willingness, strong concern about uncritical use, and barriers involving reliability, regulation, and accountability. The central challenge therefore appears to be competence and governance rather than acceptance. Evidence from automated pill-recognition technology likewise shows that uncertainty information can shape pharmacists’ trust in AI outputs [29]. Critical AI literacy extends beyond tool operation to intended use, data limitations, uncertainty, validation, bias, independent checking, escalation, and communication without transferring professional responsibility. Pharmacists must retain authority to question, override, or reject outputs. Governance is equally important. Before AI-supported medication review or pharmacotherapy decisions become routine, organizations need to define approved use cases, minimum evidence standards, local validation, data protection, monitoring, responsibility for errors, documentation, and procedures for uncertain or conflicting output [30,31]. A high relevance rating should therefore not be interpreted as readiness for unrestricted adoption. It identifies a field that participants considered highly relevant but for which clearer standards for responsible use appear necessary.

### 4.3. Assisted Suicide: Defining an Ethically Supported Professional Role

Assisted suicide combined broad professional support with lower perceived capability and barriers spanning ethics, organization, emotional burden, law, professional roles, and communication. Support for a lawful professional role should therefore not be equated with personal or organizational preparedness. Assisted-suicide-related pharmacy practice cannot be implemented as a purely technical medicines-supply procedure. It may require verification of legal requirements, accurate handling and documentation, sensitive communication, protection of confidentiality, coordination with other professionals, and management of moral disagreement. Participation must remain consistent with the applicable legal framework and with protection for conscientious non-participation, while systems must also avoid creating arbitrary barriers for eligible patients [13].

Consistent with previous pharmacy research on medical assistance in dying, the emotional-burden risk item rating and barrier profiles indicate a need for supportive structures rather than an expectation that individual pharmacists manage the burden alone [32]. Preparatory education, case-based ethical discussion, clear standard operating procedures, access to consultation, team debriefing, and referral to psychological support may all be relevant. These interventions should be evaluated for acceptability and effectiveness rather than assumed to be sufficient.

### 4.4. Implications for Research and the Development of Pharmacy Practice

The comparison suggests that willingness is only one layer of scope expansion. Responsible implementation may depend on alignment among professional legitimacy, individual capability, organizational capacity, and governance or supportive safeguards, with field-specific strategies addressing the most salient concerns. This field-specific view has consequences for professional education. POCT requires clinical interpretation and service-delivery competence; AI may require digital critical appraisal and calibrated use; assisted suicide requires legal, communicative, ethical, and reflective competence. A single generic curriculum on “new pharmacy services” would not adequately prepare practitioners for these different responsibilities. Education could instead combine a common foundation in patient assessment, documentation, interprofessional communication, implementation science, and professional accountability with field-specific modules. Comparable field-specific competency requirements have also been described for vaccination services and pharmacist prescribing [33,34,35]. These considerations cannot be assigned solely to individual pharmacists: organizational and policy infrastructures should accompany new responsibilities.

At the level of professional identity, the three fields illustrate a transition from stewardship of medicines as products to responsibility for decisions and processes surrounding medicines [2,36]. POCT places pharmacists upstream in assessment and referral. AI requires pharmacists to supervise and critically interpret algorithmic recommendations. Assisted suicide places pharmacists within a legally and ethically consequential care process. The profession’s development therefore involves not only performing more tasks, but accepting new forms of clinical, epistemic, and moral responsibility. This interpretation is heuristic rather than a validated readiness model and its applicability should be tested in future studies.

### 4.5. Strengths and Limitations

Strengths include the format of a purpose-designed interactive symposium workshop devoted to the responsible implementation of emerging pharmacy roles, recruitment through a national professional body, complete questionnaire return from all 45 event participants, and integration of structured ratings with spontaneous barrier entries. The experienced, practice-oriented sample and field-by-field comparison revealed patterns that separate topic-specific surveys might miss.

Limitations include the small, self-selected symposium sample, which was predominantly Austrian and community-pharmacy based and does not permit estimation of national prevalence, organizational readiness, or service feasibility. No formal a priori power calculation was performed. Legal scope, service maturity, remuneration, professional mix, and event participation limit external validity and qualitative transferability. The study-specific questionnaire used one item per topic-specific content area and a deliberately chosen forced-choice scale without a neutral midpoint, favoring directional judgments over neutral responses. The items underwent internal face review and pretesting for apparent relevance, clarity, readability, comprehensibility, and usability, but were not designed or validated as psychometric scales; repeated labels therefore do not establish latent constructs or measurement equivalence. Each content area relied on a single study-specific item, and non-relevance items were not fully parallel; capability therefore reflects perceived capability rather than validated self-efficacy.

Questionnaire and online responses were anonymous and unlinked, limiting integration to the group level. Brief entries could not be probed, and live visualization may have influenced subsequent discussion. Category frequencies therefore indicate salience within the submitted material, not participant prevalence or thematic saturation. The topic-specific scientific presentations and moderated discussions preceded the online barrier elicitation and were intentional components of the deliberative study setting. Accordingly, the findings represent symposium-informed judgments rather than unconditioned baseline attitudes and may reflect framing, order, and social-interaction effects. The paper questionnaire was distributed at the beginning and collected at the end, so individual item-completion times cannot be determined.

Cross-field inferential testing was restricted to the prespecified relevance comparison. Perceived capability, support, field-specific risk, and implementation-related willingness were interpreted descriptively because the corresponding items were not fully parallel. Experience groups were small, and nominal associations were nonsignificant after adjustment. The cross-sectional design precludes causal inference. Because discussions were not analyzed, claims about collective perspective development or consensus remain unsupported.

## 5. Conclusions

In this exploratory symposium sample, favorable attitudes toward expanded pharmacy roles coexisted with different activity-specific implementation patterns. POCT responses were accompanied mainly by resource and workflow concerns; AI responses by concerns about critical competence, reliability, accountability, and oversight; and assisted-suicide-related responses by legal, organizational, communicative, ethical, and emotional support needs. The post -hoc shorthand labels used to summarize these patterns are heuristic rather than validated constructs, and the findings do not establish readiness beyond this exploratory symposium sample or demonstrate service feasibility. Taken together, these findings suggest that professional support alone is an insufficient indicator of implementation readiness; future implementation research should therefore examine activity-specific combinations of professional legitimacy, individual capability, organizational delivery capacity, and governance or supportive safeguards rather than assume a uniform implementation strategy. Future studies should test these hypotheses in larger and more diverse samples using validated parallel measures and participant-linked mixed-methods data.

## Figures and Tables

**Table 1:** Participant characteristics and prior field-specific experience (*N* = 45).

Characteristic	Category	*n*	%
Practice setting	Community pharmacy	36	80.0
	Hospital/nursing home	3	6.7
	Industry/research	1	2.2
	Other	5	11.1
Country of employment	Austria	37	82.2
	Germany	7	15.6
	Other	1	2.2
Professional experience	<1 year	8	17.8
	1–3 years	5	11.1
	4–10 years	7	15.6
	11–20 years	11	24.4
	>20 years	14	31.1
Age group	18–29 years	10	22.2
	30–44 years	16	35.6
	45–59 years	16	35.6
	60+ years	3	6.7
Gender	Female	31	68.9
	Male	13	28.9
	Missing	1	2.2
Prior experience *	POCT	28	62.2
	AI	7	15.6
	Assisted suicide	12	26.7
	None of the three fields	11	24.4

* Multiple selections were possible; percentages are based on all questionnaires.

**Table 2:** Item-level questionnaire results by field and dimension with scoring on the 1–4 scale (1 = strongly disagree; 4 = strongly agree).

Field	Dimension	Agreement, *n/N* (%)	Median [IQR]	Mean	SD
POCT	Perceived capability	40/45 (88.9%)	4.0 [3.0–4.0]	3.47	0.92
	Relevance	41/44 (93.2%)	4.0 [4.0–4.0]	3.73	0.66
	Support	42/44 (95.5%)	4.0 [3.5–4.0]	3.68	0.64
	Field-specific risk *	11/45 (24.4%)	2.0 [1.0–2.0]	2.00	0.93
	Implementation-related willingness	43/45 (95.6%)	4.0 [4.0–4.0]	3.71	0.69
AI	Perceived capability	26/43 (60.5%)	3.0 [2.0–4.0]	2.74	1.03
	Relevance	42/44 (95.5%)	3.0 [3.0–4.0]	3.43	0.59
	Support	38/44 (86.4%)	4.0 [3.0–4.0]	3.36	0.84
	Field-specific risk *	36/44 (81.8%)	3.0 [3.0–4.0]	3.18	0.99
	Implementation-related willingness	31/44 (70.5%)	3.0 [2.0–4.0]	2.93	0.95
**Assisted suicide**	Perceived capability	23/42 (54.8%)	3.0 [2.0–3.0]	2.57	1.09
	Relevance	34/43 (79.1%)	3.0 [3.0–4.0]	3.14	0.80
	Support	37/44 (84.1%)	4.0 [3.0–4.0]	3.30	0.90
	Field-specific risk *	26/44 (59.1%)	3.0 [2.0–4.0]	2.70	1.05
	Implementation-related willingness	32/44 (72.7%)	4.0 [2.0–4.0]	3.16	1.12

* Risk items are shown in their original direction. Agreement therefore indicates concern that POCT may create false reassurance or reduce necessary physician consultation, that AI recommendations may be followed uncritically, or that assisted-suicide-related work may cause emotional burden. Agreement denotes response options 3 or 4. IQR, interquartile range; SD, standard deviation.

**Table 3:** Practice-informed implementation considerations derived from mixed-methods integration.

Field	Empirical Pattern	Heuristic Shorthand	Candidate Implementation Considerations
POCT	High relevance, support, perceived capability, and willingness	Endorsement-capacity gap	Workforce and time; sustainable remuneration; space and equipment; quality management; documentation; referral and treatment pathways
AI	High relevance and support; lower capability; strong concern about uncritical use	Capability–governance gap	AI literacy and critical appraisal; fit-for-purpose validation; data governance; accountability; monitoring; human oversight and escalation
Assisted suicide	Broad support and willingness; lower capability; substantial emotional-risk perception	Support-preparedness gap	Legal and role clarity; voluntary participation; communication and ethical reflection; team procedures; emotional support; continuity of lawful access

The gap labels are post -hoc heuristic interpretive summaries rather than validated constructs or a validated framework.

## Data Availability

The original contributions presented in this study are included in the article/supplementary material (https://doi.org/10.5281/zenodo.22308485, accessed on 11 September 2026). Further inquiries can be directed to the corresponding author.

## Supplementary Materials

The following supporting information can be downloaded at: https://www.mdpi.com/article/10.3390/pharmacy14060140/s1, Table S1: Paper questionnaire administered during the workshop.

## Abbreviations

The following abbreviations are used in this manuscript:

Artificial intelligence	AI
Interquartile range	IQR
Paracelsus Medical Private University	PMU
Point-of-care testing	POCT
