# Supplementary material for: Beyond the Dispensary: Pharmacists’ Perspectives on the Responsible Implementation of Emerging Clinical, Digital, and Ethically Sensitive Roles
Source: Pharmacy (Basel). 2026 Sep 21;14(6):140. doi: 10.3390/pharmacy14060140 (PMC13610308; doi:10.3390/pharmacy14060140)
Supplement: Supplementary file 1 [file pharmacy-14-00140-s001.zip › pharmacy-4513063-supplementary.pdf]

Table S1: Paper questionnaire administered during the workshop.

Personal details

**Setting**	☐Community pharmacy ☐Hospital/long-term care ☐Industry/research ☐ Other
**Country of employment**	☐ Austria ☐ Germany ☐ Other: _______________
**Years in practice**	☐ <1 ☐ 1–3 ☐ 4–10 ☐ 11–20 ☐ >20
**Age**	☐ 18-29 ☐ 30-44 ☐ 45-59 ☐ 60+
**Gender**	☐ female ☐ male ☐ other
**Prior experience**	☐Assisted suicide ☐Point-of-Care Testing (POCT) ☐Artificial intelligence in pharmacotherapy ☐ none

	**Statement**	**1 Strongly disagree**<br>**2 Rather disagree**<br>**3 Rather agree**<br>**4 Strongly agree**
**Assisted suicide**	I feel able, as part of my pharmaceutical practice, to undertake tasks related to assisted suicide competently and in accordance with legal requirements.	1☐ 2☐ 3☐ 4☐
	The work performed by pharmacists in connection with assisted suicide is relevant to current and future pharmacy practice.	1☐ 2☐ 3☐ 4☐
	I consider the involvement of pharmacists in activities related to assisted suicide to be professionally and ethically acceptable.	1☐ 2☐ 3☐ 4☐
	The emotional burden makes it more difficult for me to provide pharmaceutical services related to assisted suicide.	1☐ 2☐ 3☐ 4☐
	I am generally willing, as part of my pharmaceutical practice, to actively undertake tasks related to assisted suicide - irrespective of my current professional confidence and my personal ethical position.	1☐ 2☐ 3☐ 4☐
**Artificial intelligence in pharmacotherapy**	I feel professionally able to use artificial intelligence safely in medication reviews and to interpret its results appropriately.	1☐ 2☐ 3☐ 4☐
	I consider artificial intelligence important for future pharmacy practice and believe that it can improve the quality of medication reviews.	1☐ 2☐ 3☐ 4☐
	I support greater involvement of pharmacy professionals in the use of artificial intelligence in pharmacotherapy and medication management.	1☐ 2☐ 3☐ 4☐
	I see a risk that people may follow artificial intelligence recommendations uncritically.	1☐ 2☐ 3☐ 4☐
	I intend to use artificial intelligence to support medication reviews in my professional practice, irrespective of my current professional confidence.	1☐ 2☐ 3☐ 4☐
**Point-of-Care Testing (POCT)**	I feel professionally competent to perform POCT measurements, interpret the results correctly, and provide patients with appropriate advice on the findings.	1☐ 2☐ 3☐ 4☐
	POCT currently and in the future constitutes a relevant task in pharmacy practice and contributes to timely, patient-centred care.	1☐ 2☐ 3☐ 4☐
	I consider greater involvement of pharmacy staff in POCT and participation in public screening programmes to be appropriate.	1☐ 2☐ 3☐ 4☐
	I see a risk that this service may lead patients to attend necessary medical appointments less often, potentially leaving diseases undetected or giving patients a false sense of security.	1☐ 2☐ 3☐ 4☐
	I would consider using POCT and/or conducting screenings in my professional practice, irrespective of my current level of professional confidence.	1☐ 2☐ 3☐ 4☐

Dear Participant,

We warmly invite you to take part in our study!

The aim of this study is to systematically assess and analyse willingness to engage with evolving fields of pharmaceutical practice and the intention to implement corresponding services. The content focuses on assisted suicide, point-of-care testing (POCT), and the use of artificial intelligence in pharmacotherapy. The findings are intended to inform evidence-based improvement and implementation strategies.

Participation in this study is voluntary. The survey will be conducted once and takes approximately 8–10 minutes. The questionnaire will be provided in paper form. In addition to the questionnaire, electronic Mentimeter questions are to be answered. **Data collection is fully anonymous.** This applies both to the paper questionnaire and to the supplementary Mentimeter survey. Mentimeter is a web-based survey tool that enables anonymous polling. Mentimeter processes the collected data in compliance with the GDPR on servers within the European Union.

The collected data will be used in anonymised form exclusively for scientific purposes. Participation involves **no costs**; no remuneration is provided. You have the right to discontinue participation at any time without giving reasons and without any resulting disadvantage. As the data are collected anonymously and cannot be linked to individual persons, data already submitted cannot be withdrawn after completion of data collection. The data will be stored in password-protected form on the server of Paracelsus Medical University (PMU) in accordance with data protection requirements.
PMU Data Protection Officer: datenschutz@pmu.ac.at. You have the right to lodge a complaint with the Austrian Data Protection Authority regarding the handling of your data (www.dsb.gv.at; email: dsb@dsb.gv.at).

The anonymously collected data will be accessed exclusively by the research team (Mag. pharm. E. DUSINOVIC, Mag. pharm. C. SIMON, Pharmacist C. MOSCH) and, after expiry of the retention period, will be properly destroyed in accordance with the applicable scientific standards.

For questions about the study, please contact the study director (Priv.-Doz. Dr. Olaf Rose, PharmD) at: olaf.rose@pmu.ac.at

Informed consent statement

By actively submitting the completed questionnaire, you declare your voluntary participation in the study and consent to the use of the **fully anonymous** data for scientific purposes. You also confirm that you have read and understood the information above.
